# Expectant Management of a Twin Pregnancy with Complete Hydatidiform Mole and Coexistent Normal Fetus

**DOI:** 10.1155/2019/8737080

**Published:** 2019-10-07

**Authors:** Colin Johnson, Caroline Davitt, Rachel Harrison, Meredith Cruz

**Affiliations:** Department of Obstetrics and Gynecology, Medical College of Wisconsin, Milwaukee, WI, USA

## Abstract

Twin pregnancies complicated by complete hydatidiform mole coexisting with a viable fetus are rare and may result in significant complications. We describe the expectant management and our surgical approach in a 27-year-old Rh-negative woman presenting with recurrent episodes of vaginal bleeding and a twin pregnancy consisting of a molar pregnancy coexisting with a normal fetus. Inpatient management was undertaken with close maternal and fetal monitoring until cesarean delivery of a healthy female infant and histopathologically confirmed complete hydatidiform molar pregnancy (karyotype 46XX) at 34 weeks with no evidence of malignancy.

## 1. Introduction

Complete hydatidiform molar pregnancy is a premalignant condition typically resulting from the anomalous fertilization with diploid expression of the paternal genome and is characterized by trophoblastic hyperplasia and abnormal chorionic villi [1, 2]. Twin pregnancies consisting of a complete hydatidiform mole coexisting with a normally developing fetus (CHMCF) are exceedingly rare, estimated at one in 20,000–100,000 pregnancies [3]. Only 40% of CHMCF result in live births [4]. Despite traditional recommendation for termination of pregnancy for maternal safety, evidence shows the risk of gestational trophoblastic neoplasia (GTN) is not significantly increased with continuation of pregnancy [3, 5]. Although there are considerable risks to consider when continuing the pregnancy, this case introduces the possibility of expectant management to maintain the normally developing fetus when desired by the patient and family. We illustrate strategies for successful management of CHMCF in an Rh-negative mother despite frequent second trimester vaginal bleeding with delivery of a viable fetus at 34 weeks gestation.

## 2. Case Presentation

A healthy 27-year-old woman, gravida 4 para 3, presented with vaginal bleeding at 16 weeks gestation after a normal first trimester ultrasound. Ultrasound was performed upon presentation that demonstrated a twin pregnancy consisting of a suspected CHMCF. The suspected molar pregnancy extended from left mid uterus to fundus and measured 16.1 cm × 11.1 cm × 16 cm. No evidence of abruption was noted. Chest X-ray at the time showed no evidence of metastasis. At this time, she was offered termination of pregnancy and declined. She additionally declined all prenatal genetic testing, including amniocentesis, at the time of diagnosis. She continued to experience episodic vaginal bleeding with brown spotting between episodes. At 21 weeks, human chorionic gonadotropin (HCG) was drawn and was 226,910 mIU/mL (normal 21 weeks gestation range: 4,213–42,692) [6]. She received a course of betamethasone at 23 weeks 3 days.

**Figure 1:**
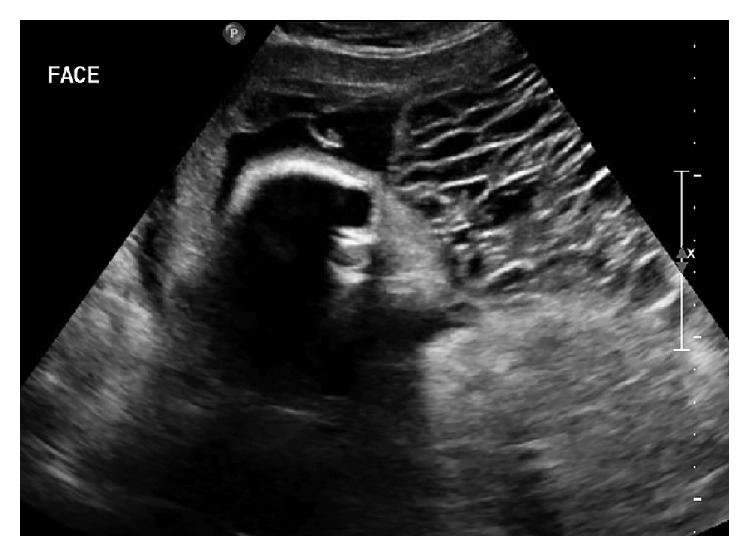
Ultrasound appearance of the fetus next to the molar component of the pregnancy at 26 weeks.

**Figure 2:**
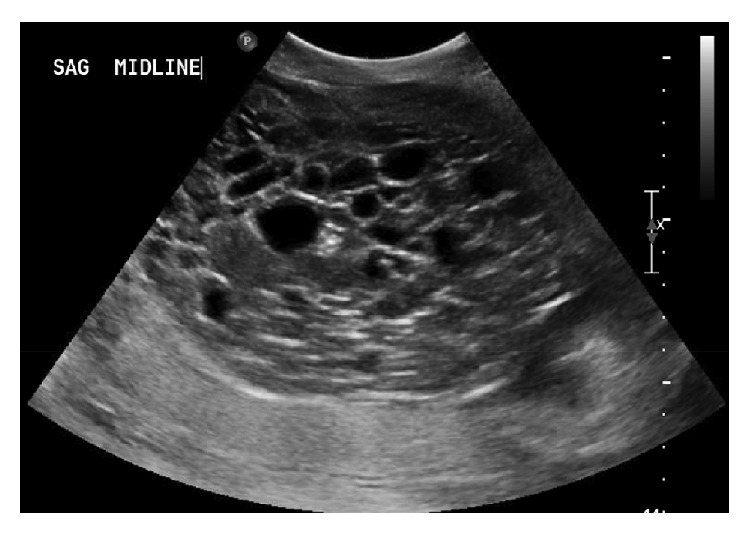
Ultrasound appearance of the molar component of the pregnancy.

At 24 weeks 6 days she was transferred to our institution due to persistent vaginal bleeding. Ultrasound at that time demonstrated an appropriately grown fetus with a suspected complete hydatidiform mole. The sagittal view of the fundal molar pregnancy adjacent to the fetal face is shown in Figure 1, and the molar pregnancy alone is shown in Figure 2. Pre-eclampsia work-up, thyroid studies, complete blood count, coagulation panel, and chest X-ray were performed upon admission and were within normal limits. HCG at this time had decreased to 190,369 (normal range at 25 weeks gestation: 3,847–53,383) [6]. Although she was not actively bleeding on admission, due to the previously noted frequent episodes of vaginal bleeding, the decision was made for inpatient management until delivery at 34 weeks by repeat cesarean section or earlier for maternal or fetal indications. Gynecologic oncology was consulted on admission.

During her hospitalization, episodes of vaginal bleeding of roughly 100–300 mL occurred every 2–5 days from 24 weeks 6 days until her last episode of bleeding at 27 weeks 3 days. A complete blood count (CBC) and Kleihaur-Betke (KB) were drawn with each episode of bleeding and Rh immunoglobulin was administered accordingly due to maternal Rh-negative blood type. As an inpatient she received routine prenatal care including gestational diabetes screening and Tdap administration. She had an active type and screen at all times, and received a second course of betamethasone at 33 weeks 5 days in preparation for delivery. Notably, fetal monitoring was initially performed three times per day due to frequent bleeding episodes and was decreased to once per day after the bleeding resolved.

**Figure 3:**
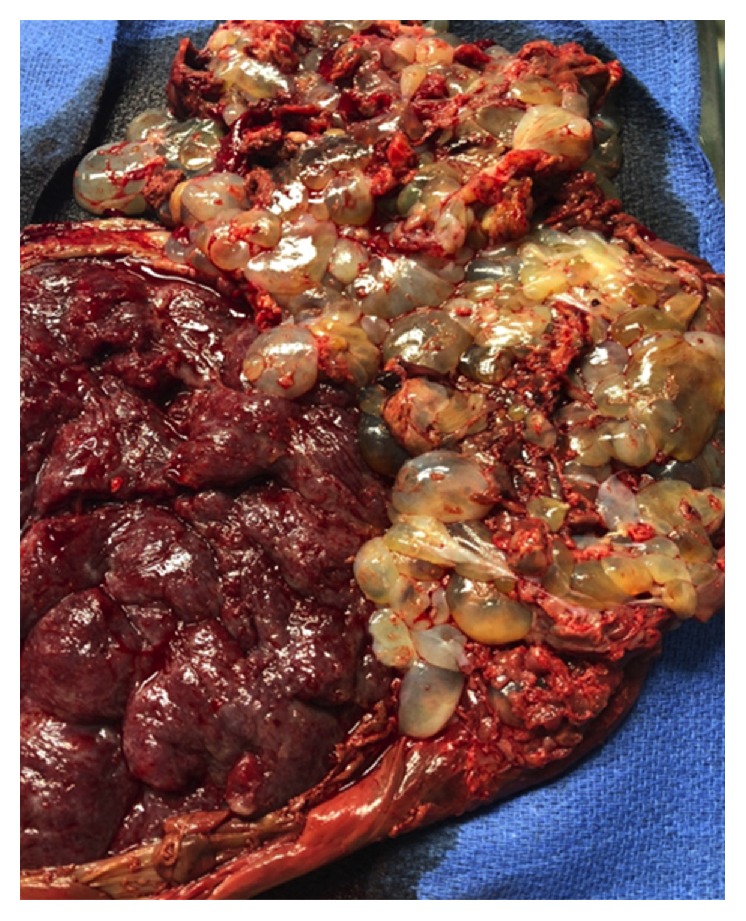
Placenta of normal pregnancy adjacent to complete molar pregnancy at delivery.

At 34 weeks 1 day she underwent a repeat cesarean section with delivery of an appropriately grown 2150 g female infant with Apgar scores of 9 and 9 at one and five minutes, respectively. Preparations were in place for a hysterectomy if indicated, and the surgery was performed in the main hospital operating room due to proximity to the intensive care unit and blood bank in the event of massive transfusion. Copious amounts of molar tissue spontaneously delivered with the placental tissue (Figure 3). The remaining placental and molar tissue was evacuated from the uterine cavity using laparotomy sponges repeatedly due to the significant quantity and fragility of the molar tissue. Gentle curettage was then performed through the open hysterotomy via a large banjo curette to remove the remainder of molar pregnancy from the uterine fundus. Estimated blood loss was 700 mL. She did not require a blood transfusion.

Her postpartum course was uncomplicated with discharge home on postpartum day three. Pathology confirmed the presence of complete hydatidiform mole with karyotype 46XX. Weekly follow up demonstrated appropriately declining HCG levels. Her last HCG was 6.1 mIU/mL roughly 3 months after delivery. No other evidence of malignancy was found.

## 3. Discussion

CHMCF is an exceptionally rare condition that is frequently accompanied by complications including vaginal bleeding, hyperemesis gravidarum, hyperthyroidism, thromboembolic disease, intrauterine fetal death, and pre-eclampsia [3–5, 7]. Due to the severity of complications, expectant management of a desired continued pregnancy is not without substantial risks. Despite these risks, we aim to strengthen practitioner confidence in expectant management of such patients by sharing our experience and outlining a treatment plan in high resource settings.

Patient counseling on the risks of complications and malignancy should be emphasized in a multidisciplinary setting with Gynecologic Oncologists and Maternal-Fetal Medicine specialists. If expectant management is chosen, patients may be reassured that several studies show similar risk of GTN with the continuation of a CHMCF pregnancy compared to a first trimester termination [3, 5]. However, due to the high-risk nature of expectant management of CHMCF, extremely close surveillance is strongly recommended—this may include hospitalization in some circumstances, particularly in those with vaginal bleeding. Postpartum management for HCG monitoring for GTN risk assessment is also essential.

Suksai et al. demonstrated that antenatal complications (especially pre-eclampsia, hyperthyroidism, and hyperemesis gravidarum) were associated with adverse perinatal events for those with CHMCF [4]. Low initial HCG level less than 400,000 mIU/mL was the best predictor of live births in these patients in a logistic regression prediction model [4]. Therefore, they suggest that patients with an initial HCG level less than 400,000 mIU/mL with no antepartum complications are appropriate candidates for pregnancy continuation. Our patient exhibited consistent sub-threshold HCG and did not experience any additional antepartum complications after vaginal bleeding ceased during her 27^th^ week of gestation.

An essential aspect in the successful management of this case was the high resource setting in which the patient was cared for that included the following: a level four neonatal intensive care unit (NICU), a large hospital system with the availability of a variety of subspecialists, availability of massive transfusion, and expertise in high-risk pregnancy. In addition to interdisciplinary management, preparedness for all outcomes in an optimally equipped operative room at the time of delivery and aggressive resection of the hydatidiform molar tissue was imperative. The NICU team was present and evaluated the live infant upon delivery. In accordance with the National Cancer Institute's recommendations, the uterus was cleaned with multiple passages of laparotomy sponges, and a large banjo curette was used to ensure complete evacuation of molar tissues [8].The significant decline in HCG levels after delivery demonstrates the effectiveness of curettage.

Due to the low number of pregnancies continued into the third trimester with CHMCF, it is important to acknowledge that situations may arise in which it may not be safe for the mother and/or the viable fetus to continue the pregnancy. During the course of our patient's care, delivery was contemplated multiple times due to episodes of vaginal bleeding which fortunately rapidly resolved on each occurrence and were not associated with any fetal heart rate changes or maternal vital sign changes. In those who experience continuous vaginal bleeding, bleeding with maternal instability or fetal heart rate changes, or other complications such as pre-eclampsia or intrauterine fetal demise, delivery should be expedited. The delivery timing of such patients must be a multi-disciplinary and multi-provider decision in conjunction with the patient and family, with flexibility contingent upon the clinical situation. This case illustrates our successful clinical and operative strategies for the safe expectant management and delivery of a viable fetus coexisting with complete hydatidiform molar pregnancy into the third trimester in a high resource setting.
